# Supplementary material for: Multi-project wafer runs for electronic graphene devices in the European 2D-Experimental Pilot Line project
Source: Nat Commun. 2025 Feb 6;16:1417. doi: 10.1038/s41467-025-56357-0 (PMC11802748; doi:10.1038/s41467-025-56357-0)
Supplement: Supplementary file 1 — Supplementary Information [file 41467_2025_56357_MOESM1_ESM.pdf]

Supplementary Information

Multi-project wafer runs for electronic graphene devices in the European 2D-Experimental Pilot Line project

Bárbara Canto,[1] Martin Otto,[1] Arantxa Maestre,[2, &] Alba Centeno,[2] Amaia Zurutuza,[2] Bianca Robertz,[1] Eros Reato,[3] Bartos Chmielak,[1] Stefanie L. Stoll,[1] Andreas Hemmetter,[1,3] Florian Schlachter,[1] Lisa Ehlert,[1] Sha Li,[1,§] Daniel Neumaier,[1,4] Gordon Rinke,[1] Zhenxing Wang,[1,*] Max C. Lemme[1,3*]

[1] AMO GmbH, Otto-Blumenthal-Str. 25, 52074 Aachen, Germany

[2] Graphenea S.A., Paseo Mikeletegi 83, 20009 San Sebastián, Spain

[3] RWTH Aachen University, Chair of Electronic Devices, Otto-Blumenthal-Str. 2, 52074 Aachen, Germany

[4] University of Wuppertal, Chair of Smart Sensor Systems, Lise-Meitner-Str. 13, 42119 Wuppertal, Germany

[*] Correspondence: wang@amo.de, lemme@amo.de

Current Address:

[&] ASM International, Kapeldreef 75, 3001 Leuven, Belgium

[§] Heraeus Precious Metals GmbH & Co. KG Herauesstr. 12-14, 63450, Hanau, Germany

Supplementary Note 1 - Raman Analysis

Raman maps were measured at the center and the edge of the wafer (see Methods in main paper). Typical Raman spectra, i.e., spectra with the median value of intensity ratio of the D and G peaks (I_D/I_G) for each respective map (after transfer and after fabrication), measured at the center of each wafer, are shown in Supplementary Figure 1.1 for the two runs. For MPW run 1, the spectra after fabrication were measured on devices without encapsulation because it was not practical to measure the encapsulated graphene. The encapsulation was 200 nm thick for the MPW run 1 and severely decreased the intensity of the Raman signal. For MPW run 1, the D peak at around 1350 cm^{-1} is very small, indicating high graphene quality.[1] More importantly, there is no noticeable change in the D peak intensity before and after fabrication. For MPW run 3, on the other hand, there is an emergence of additional peaks and an increase of the D peak after the encapsulation. Typical spectra for all the fabrication steps are shown in Supplementary Figure 1.2a for MPW run 3. It is evident that only the encapsulation by ALD causes a significant increase in the D peak and additional Raman peaks in the vicinity of the D and G peaks. These peaks can be associated with amorphous carbon (a-C peak).[2,3] However, when fitting the overlapping peaks in this region of the spectrum, it is difficult to differentiate between the D peak of graphene and the peaks attributed to the amorphous carbon. Because of this, it is quite possible that we are overestimating the amplitude of the D peak. The main cause for these additional peaks in MPW run 3 is the deposition method of the encapsulation in conjunction with the presence of photoresist residue on the graphene. For MPW run 1, the encapsulation was deposited by e-beam evaporation and the wafer remained at a temperature of less than 50 °C. For MPW run 3, the encapsulation was deposited by ALD at a chuck temperature of 300 °C, which corresponds to a wafer temperature of around 200 °C. This is hot enough to burn the resist residue from the graphene patterning step. The reason we believe this is related to burned resist and not to the ALD process itself, will be discussed here. The encapsulation process that was used (more details in the Methods section of the main paper) is a combination of the thermal ALD and PEALD process at 300 °C.

However, these peaks only appear if the graphene has been subjected to lithography and RIE prior to ALD, i.e. there is resist residue present on the graphene. For example, Supplementary Figure 1.2b shows spectra for three different scenarios before and after encapsulation: first, the encapsulation by e-beam evaporation at less than 50 °C from MPW run 1, second, the encapsulation by ALD at 300 °C and third, the encapsulation of graphene by ALD at 300 °C on a test wafer without prior lithography and RIE on the graphene. It is clear in the spectra from Supplementary Figure 1.2b that the a-C peaks only appear for the combination of higher temperatures and prior lithography, leading us to believe that burning of resist residue is responsible for the appearance of these peaks.

The statistics of I_D/I_G and the full width at half maximum of the 2D peaks, FWHM(2D), of the Raman maps are shown as box plots for the two runs in Supplementary Figure 1.3a and 1.3b, respectively, after transfer and after fabrication for MPW run 1 and for MPW run 3. Each box contains the values of both maps for each displayed fabrication step and wafer: the map from the center of the wafer and the one from the edge (details in Methods section of the main text). I_D/I_G increases from 0.04 to 0.07 after fabrication for MPW run 1 and from 0.02 to 0.35 for MPW run 3. The stronger increase of this value for the MPW run 3 probably occurred due to the presence of resist residue.

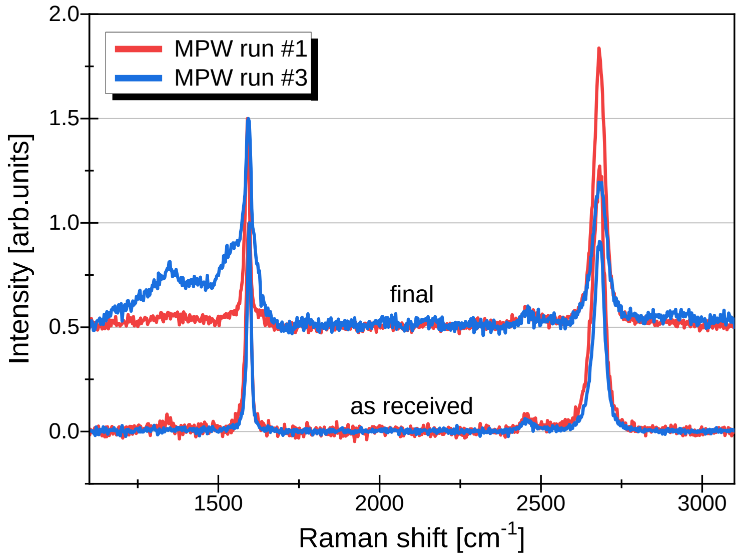

Supplementary Figure 1.1 | Typical Spectra for the MPW run # 1 and MPW run # 3 wafers, as received and after fabrication.

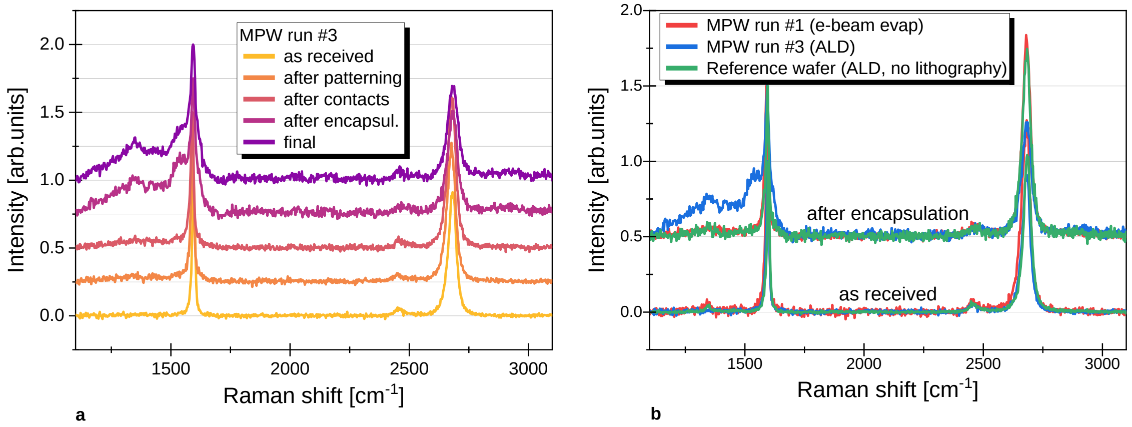

Supplementary Figure 1.2 | Raman spectra for a-C analysis, comparison tests. **a** Typical Raman spectrum for the wafer fabricated in the MPW run 3 in every step of fabrication. **b** Comparison between one reference wafer without lithography and RIE etching on graphene and the wafers from MPW run 1 and MPW run 3 as received and after ALD deposition.

The box plots for FWHM(2D) also show that the main increase in value occurs after encapsulating the graphene (reminder: MPW run 1 was measured without encapsulation). The FWHM(2D) is a measure for nanoscale strain variations. The FWHM(2D) depends not only on the quality of the graphene but also on the smoothness of the substrate and the interactions of the graphene with the substrate and the dielectric environment encapsulation.[4,5] A low FWHM(2D) corresponds to small strain variations and ultimately enables high carrier mobilities.[4,6–8] The values obtained here are higher than what is usually reported in literature.[4,7,9,10] The main reason for this is that FWHM(2D) values are usually reported for graphene transferred to clean, thermally grown SiO_2, which is smoother and cleaner than our surfaces, i.e. Al_2O_3 deposited by ALD that has already been subjected to three lithography steps.[4] We also used a Voigt profile to fit the 2D peak, which is a better fit than a Lorentzian profile but also leads to slightly larger peak widths compared to the more commonly used Lorentzian profile.[11] There is a significant increase in peak width after encapsulation. This can be attributed to the higher degree of conformality of the graphene to the rough substrate and the confinement of the graphene between two solid interfaces.[4] The median values of FWHM(2D) after transfer for MPW run 1 are around 45 cm^{-2}, much higher than expected and, more significantly, higher even than the values after device fabrication. This aspect needs further investigation. The other process steps have no direct effect on the D-peak of the FWHM(2D) but they do slightly affect the peak positions of the G and 2D peaks, which are related to strain and doping of the graphene.[7] The Supplementary Figure 1.3c shows the frequency of 2D peak position as a function of the position of the G-peak for the two runs. It is possible to notice that the doping has slightly changed during the fabrication process and the strain is higher for the MPW run 3 than for the MPW run 1. The black line represents the shifts in position due to doping of the graphene.[12]

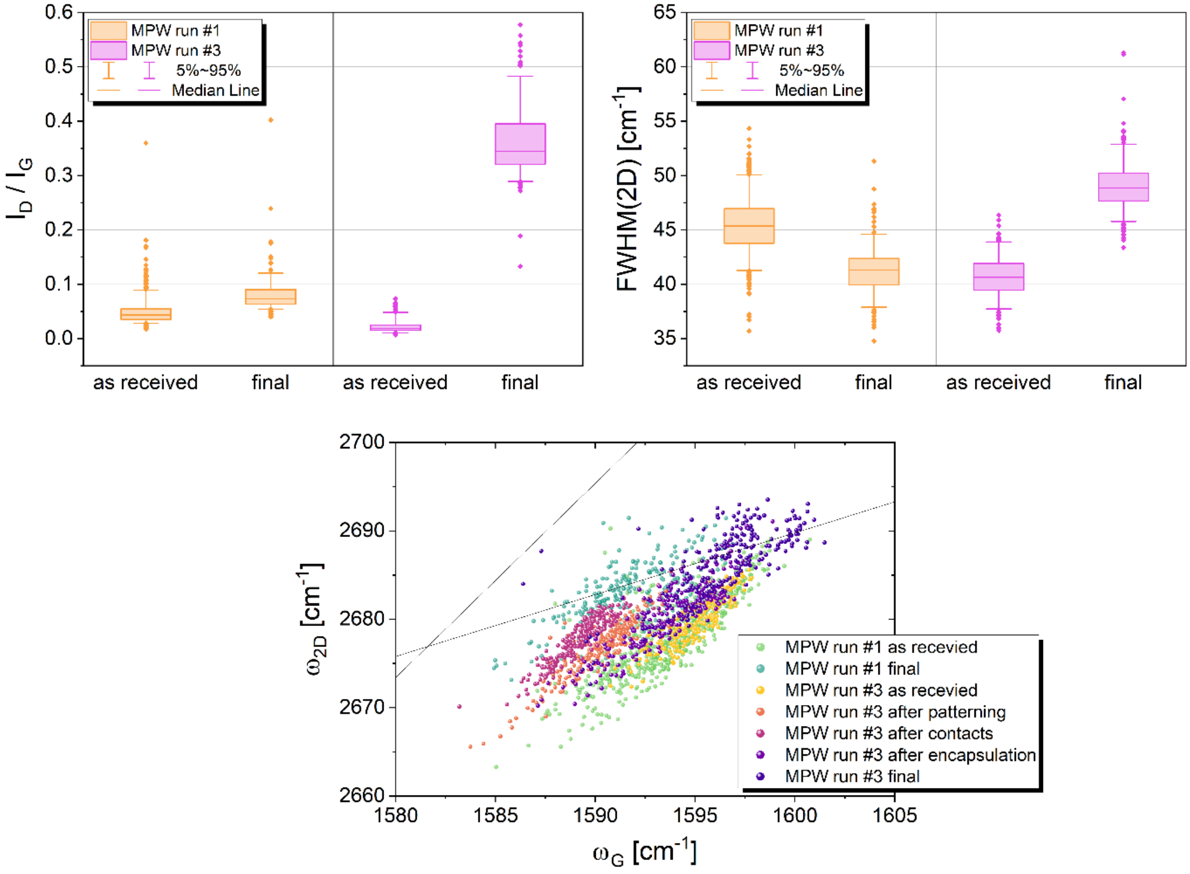

Supplementary Figure 1.3 | Raman analysis of the wafers from MPW run 1 and 3. a Statistical analysis of the I_D/I_G and **b** full width at half maximum (FWHM) of the 2D peak. **c** Scatter plot of the 2D peak position as a function of the position of the G-peak (in frequencies).

Supplementary Note 2 - Dielectric constant of the Al_2O_3 used as the back gate oxide

To characterize the dielectric properties of the Al_2O_3 used as a back gate dielectric for both MPW runs, we fabricated metal-oxide-semiconductor capacitors on separate samples. We removed the native oxide on a p-doped silicon substrate in buffered oxide etchant. The oxide removal was verified using an ellipsometer thin film measurement before and after the etching process. A 21.4 nm thick layer of Al_2O_3 was then deposited onto the bare silicon substrate using a PEALD process at 300 °C with a TMA precursor. We used negative photolithography to define circular structures with two different areas, 0.32 and 1.28 mm^2. Al/Ti (35/15 nm) contacts were deposited by e-beam evaporation, followed by lift-off. A schematic cross-section of the material stack is shown in Supplementary Figure 2.1a. Electrical measurements were performed in ambient conditions in a Cascade probe station. An AC signal with 25 mV amplitude was applied between the top circular electrode and the p-doped substrate with a frequency sweep between 100 Hz and 1 MHz. We used a *C*-G model consisting of parallel connected resistor with conductance of *G* (representing the losses in the material) and capacitor with capacitance of *C* (Supplementary Figure 2.1b)[13] to characterize the frequency response of the devices under test. The measurement is repeated on five different devices for each device area (Supplementary Figure 2.1c). From the capacitance and conductance data we calculated the complex relative permittivity, resulting in a dielectric constant of 7.3 with a deviation of 0.72 for the low frequency regime. This is the value we used for the mobility calculation in the MPW runs.

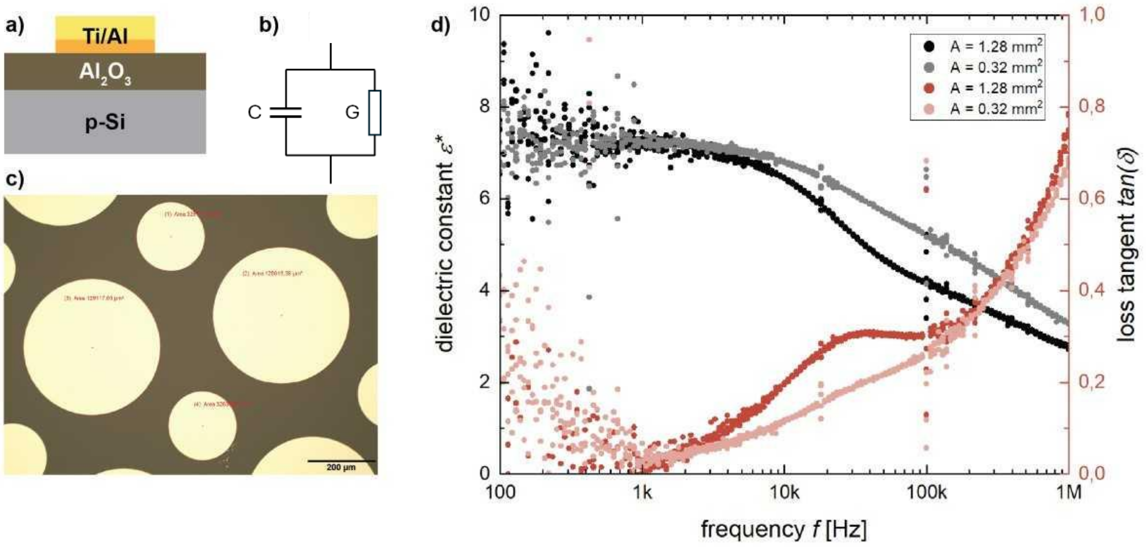

Supplementary Figure 2.1 | Dielectric constant calculation by basic capacitors. a Optical microscopy of the capacitors and **b** Schematic drawing of the devices. **c** Graphic of the dielectric constant and loss tangent for two different capacitor areas.

Supplementary Note 3 - Contact resistance calculation

As mentioned in the main text, the TLM method is not necessarily a reliable method for the extraction of the contact resistance. For the TLM method to be reliable, certain conditions need to be met, one of them being that R_c, R_{sh} and the channel width w remain constant for all channels. When this is not the case, the resulting extraction values of R_c and R_{sh} from the linear fit are no longer trustworthy. Even small fluctuations in the actual values can lead to extremely large errors in the extracted values.[14,15] In graphene, these fluctuations can be particularly large, for example due to mechanical damage (cracks or holes) in the graphene, which leads to high variability of the channel resistances.

In particular, the reliability of the contact resistance extraction at the charge neutrality point (CNP) is heavily affected by the presence of a p-p$^+$ step at the metal-graphene contact, which introduces an additional series junction resistance (R_{JUN}) that is not accounted for in the TLM model. Therefore, the incorrect modeling leads to a large number of negative values that have no physical meaning. For this reason, the values extracted at high negative bias are most reliable, where the effect of R_{JUN} is negligible[16].

Shorter channel lengths (for example less than 100 nm) can alleviate this problem, however, making shorter channels would have required using either stepper lithography or e-beam lithography. Here, we had the constraint to use contact lithography to make this process accessible to customers willing to pay only a specific price for chips made in a MPW run of the 2D -EPL. Fabricating channel lengths of 100 nm or less by e-beam lithography would have been prohibitively expensive for these customers, especially those who only required very large structures, e.g. for sensing applications.

During the preparation of the manuscript, we discussed how to present our TLM data in a more precise way. Since we (expectedly) obtained also negative values due the imprecision of the TLM method, we decided to include only the positive values in the manuscript. These were also reported

to the MPW run customers. This means that negative values of R_c, caused by large uncertainties of the fit, were deleted. This leads to a conservative median value of R_c that is too high and to sigma values that are too low. Supplementary Figure 3.1 shows the entire set of TLM plots and histograms of R_c and R_c plotted over ns for both MPW runs with negative values. Approximately half of the values for R_c are negative. The median R_c values of MPW runs 1 and 3 are 438 and 619 Ohm µm, respectively, but the sigma values are so high (4918 and 6609 Ohm µm) that the median values are not trustworthy. Supplementary Figure 3.2 shows the same data set of TLM plots with the negative data ignored (the data set used in Table 1).

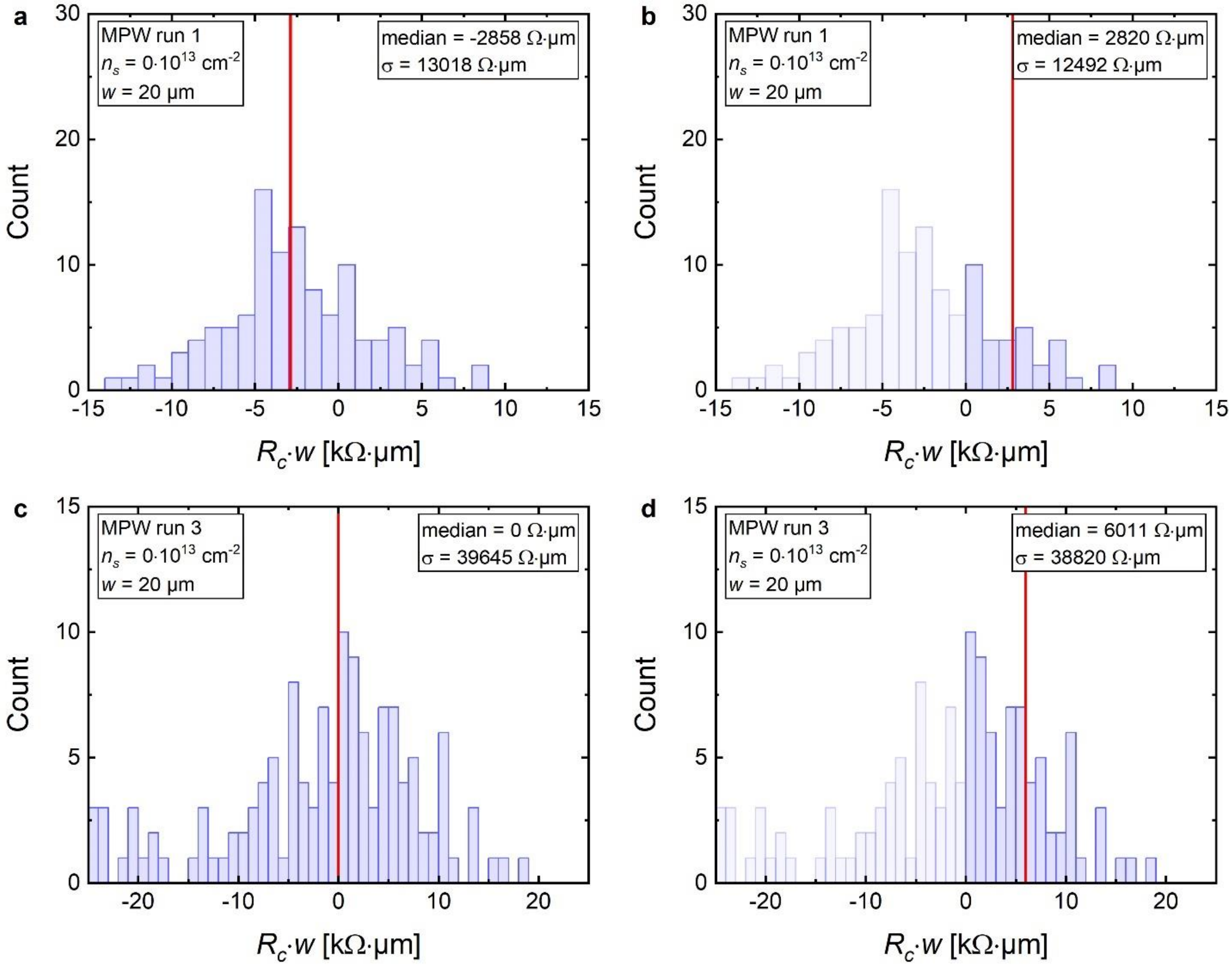

Supplementary Figure 3.1 | Histograms of the R_C·w with and without deleting the negative values for n_s = 0 cm^{-2}. **a** Histogram for MPW run 1 without deleting the negative data. **b** Histogram for MPW run 1 with deleted negative data. **c** Histogram for MPW run 3 without deleting the negative data. **d** Histogram for MPW run 3 with deleted negative data.

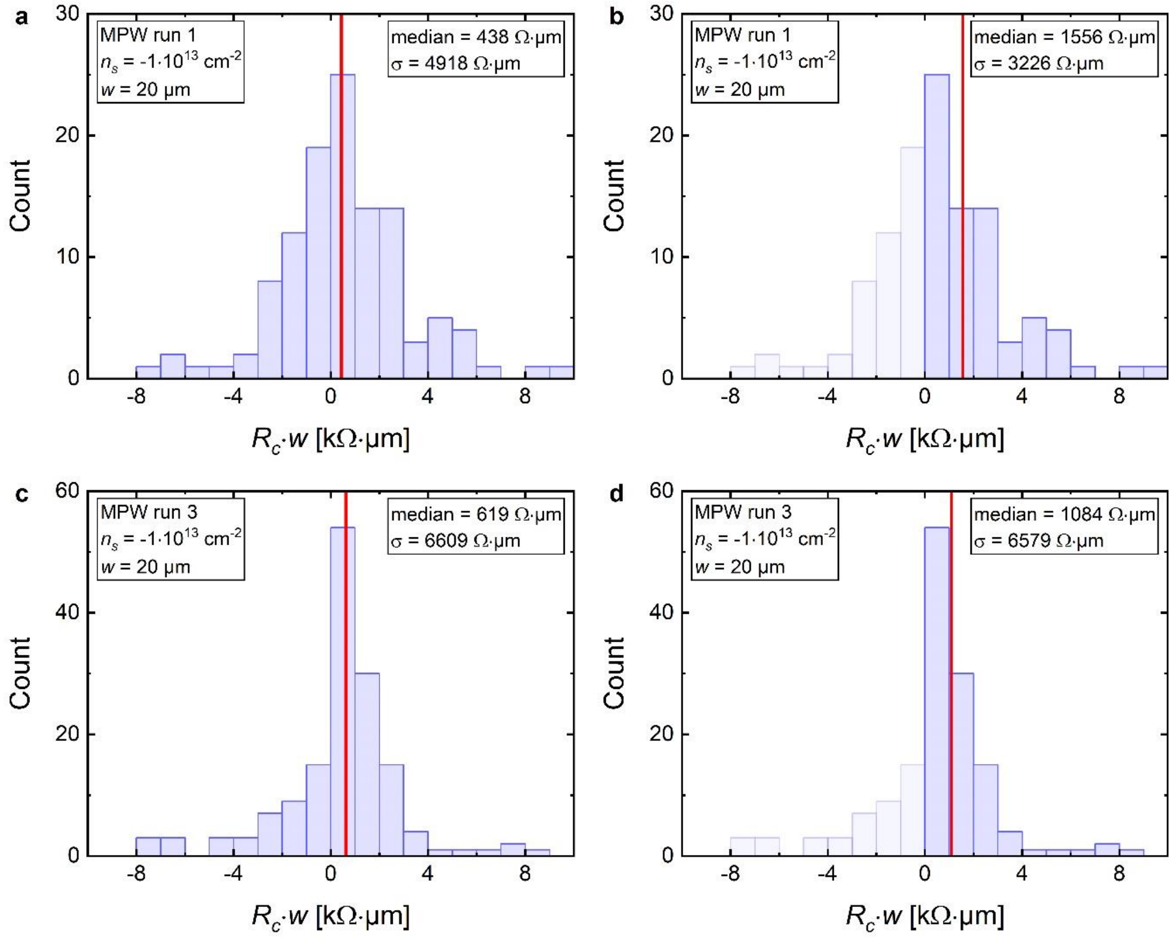

Supplementary Figure 3.2 | Histograms of the R_C·w with and without deleting the negative values for n_s = -1·10^{13} cm^{-2}. a Histogram for MPW run 1 without deleting the negative data. **b** Histogram for MPW run 1 with deleted negative data. **c** Histogram for MPW run 3 without deleting the negative data. **d** Histogram for MPW run 3 with deleted negative data.

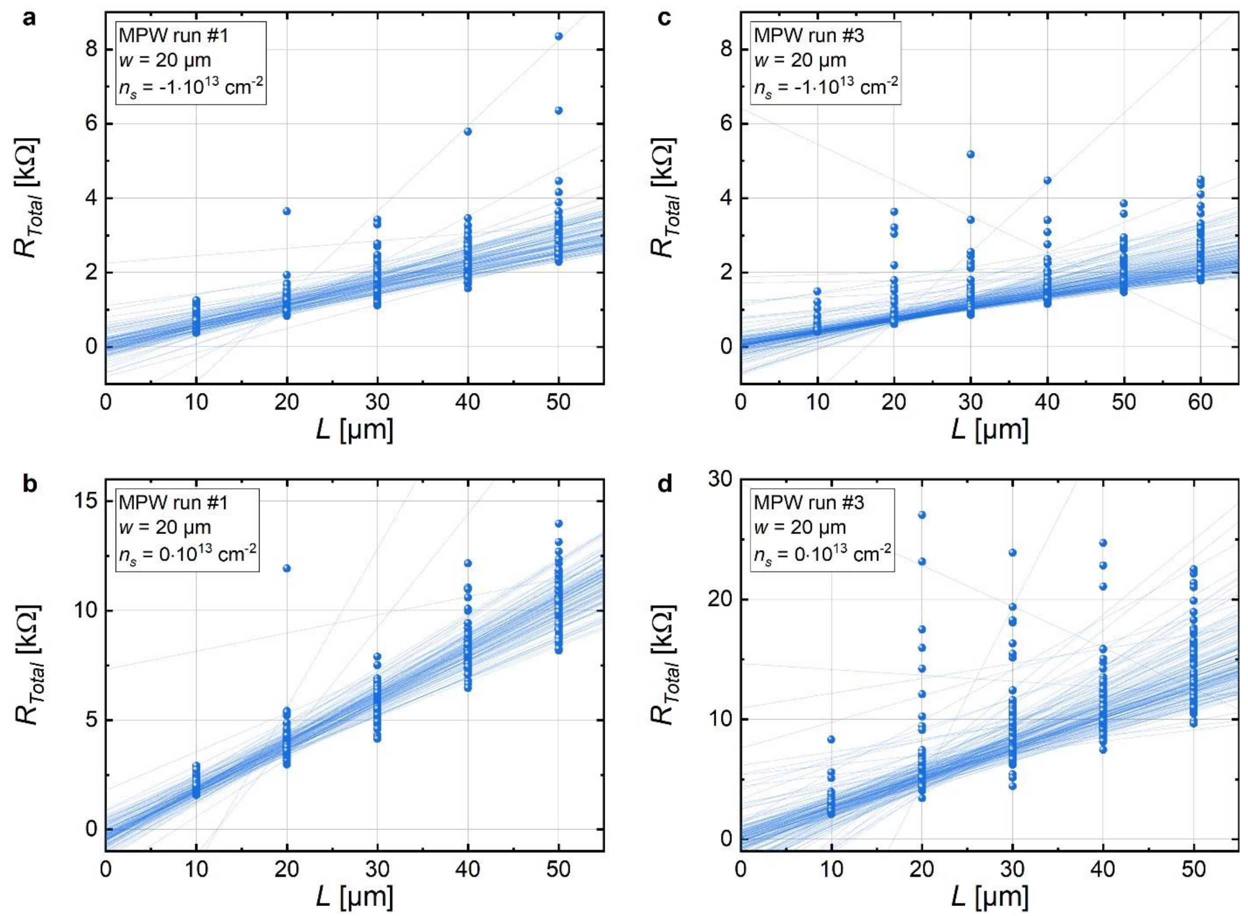

Supplementary Figure 3.3 | TLM plots for both MPW runs. a MPW run 1 at $n_s = 1{\cdot}10^{13}$ cm^{-2}. **b** MPW run 1 at n_s =0. **c** MPW run 3 at $n_s = 1{\cdot}10^{13}$ cm^{-2}. **d** MPW run 1 at n_s =0**.**

Supplementary Note 4 - Cracks in the encapsulation layer (MPW run 1)

The cracks that are mentioned in the main text were observed in the Al_2O_3 encapsulation layer and only in the regions on top of large areas of graphene, whenever the covered graphene area was hundreds of μm^2 large. Therefore, there were no cracks in the devices we measured during the MPW runs. Supplementary Figure 4.1a shows an optical microscopy of one example of the cracks. Supplementary Figure 3.1b shows an AFM image of a crack, allowing us to measure the depth of the crack. The crack extends into the graphene layer destroying this as well. In some cases, part of the dielectric layer was also removed as can be seen in Supplementary Figure 4.1c. The crack is 220 nm deep and the Al_2O_3 encapsulation layer is 200 nm thick (Supplementary Figure 4.1d).

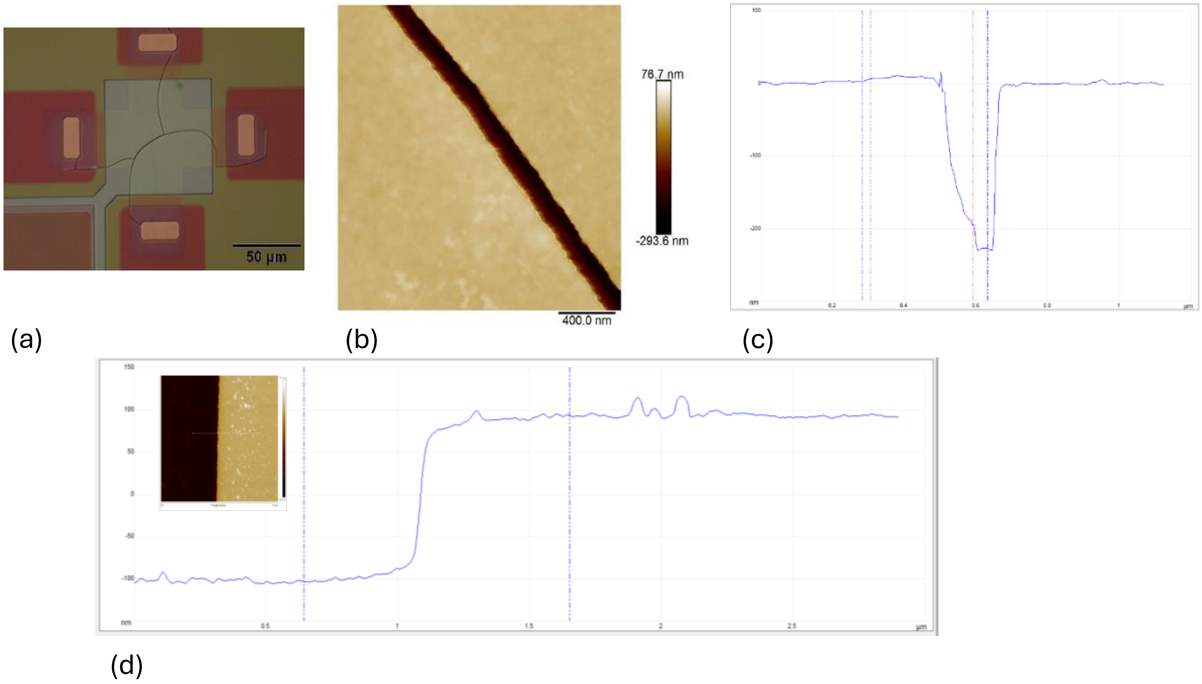

Supplementary Figure 4.1 | Cracks in the encapsulation layer of the MPW run 1. a Optical microscopy showing one device with cracks. **b** AFM image showing a crack. **c** Measurement of the depth of the crack,

red lines have 200 nm (thickness of the encapsulation layer). Blue lines are 220 nm. (d) thickness measurement of the encapsulation layer thickness.

Supplementary References

1. Malard, L. M., Pimenta, M. A., Dresselhaus, G. & Dresselhaus, M. S. Raman spectroscopy in graphene. *Physics Reports* **473**, 51–87 (2009).
2. Hong, J. *et al.* Origin of New Broad Raman D and G Peaks in Annealed Graphene. *Sci Rep* **3**, 2700 (2013).
3. Gong, C. *et al.* Rapid Selective Etching of PMMA Residues from Transferred Graphene by Carbon Dioxide. *J. Phys. Chem. C* **117**, 23000–23008 (2013).
4. Banszerus, L. *et al.* Ultrahigh-mobility graphene devices from chemical vapor deposition on reusable copper. *Sci. Adv.* **1**, e1500222 (2015).
5. Finney, N. R. *et al.* Tunable crystal symmetry in graphene–boron nitride heterostructures with coexisting moiré superlattices. *Nat. Nanotechnol.* **14**, 1029–1034 (2019).
6. Couto, N. J. G. *et al.* Random Strain Fluctuations as Dominant Disorder Source for High-Quality On-Substrate Graphene Devices. *Phys. Rev. X* **4**, 041019 (2014).
7. Canto, B. *et al.* Plasma-Enhanced Atomic Layer Deposition of Al_2O_3 on Graphene Using Monolayer hBN as Interfacial Layer. *Adv Materials Technologies* **6**, 2100489 (2021).
8. Neumann, C. *et al.* Raman spectroscopy as probe of nanometre-scale strain variations in graphene. *Nat Commun* **6**, 8429 (2015).
9. Lee, J. E., Ahn, G., Shim, J., Lee, Y. S. & Ryu, S. Optical separation of mechanical strain from charge doping in graphene. *Nature Communications* **3**, 1024 (2022).
10. Li, M. *et al.* Wafer-Scale Graphene Growth on Si/SiO2 Substrates via Metal-Free Chemical Vapor Deposition. *ACS Applied Nano Materials* **6**, 10817 (2023).
11. Bradley, M. S. Lineshapes in IR and Raman Spectroscopy: A Primer. *Spectroscopy* **30**, 42–46 (2015).
12. Gao, L. *et al.* Repeated growth and bubbling transfer of graphene with millimeter-size single-crystal grains using platinum. *Nature Communications* **3**, 699 (2012).

13. Basics of Measuring the Dielectric Properties of Materials. https://www.keysight.com/us/en/assets/7018-01284/application-notes/5989-2589.pdf.
14. Gutai, L. Statistical modeling of transmission line model test structures. I. The effect of inhomogeneities on the extracted contact parameters. *IEEE Trans. Electron Devices* **37**, 2350–2360 (1990).
15. Haw-Jye Ueng, Janes, D. B. & Webb, K. J. Error analysis leading to design criteria for transmission line model characterization of ohmic contacts. *IEEE Trans. Electron Devices* **48**, 758–766 (2001).
16. Venica, S. *et al.* On the Adequacy of the Transmission Line Model to Describe the Graphene–Metal Contact Resistance. *IEEE Transactions on Electron Devices* **65**, 1589–1596 (2018).
